# Intranasal *Chlamydia pneumoniae* Infection Induces Neuroinflammation and Cognitive Decline in Mice

**DOI:** 10.1002/alz.083564

**Published:** 2025-01-03

**Authors:** Lalita Subedi, Daisy Martinon, Jean‐Philippe Vit, Dieu‐Trang Fuchs, Malcolm E. Lane, Marianne Mita, Shuang Chen, Bhakta Prasad Gaire, Yosef Koronyo, Moshe Arditi, Maya Koronyo‐Hamaoui, Timothy R Crother

**Affiliations:** ^1^ Department of Biomedical Sciences, Infectious and Immunologic Diseases Research Center, Cedars‐Sinai Medical Center, Los Angeles, CA USA; ^2^ Cedar Sinai Medical Center, Los Angeles, CA USA; ^3^ Cedar Sinai Medical center, Los Angeles, CA USA; ^4^ Cedar SInai Medical center, Los Angeles, CA USA; ^5^ Department of Pediatrics, Cedar Sinai Medical Center, Los angeles, CA USA; ^6^ Department of Neurosurgery, Maxine Dunitz Neurosurgical Research Institute, Cedars‐Sinai Medical Center, Los Angeles, CA USA; ^7^ Cedars Sinai Medical Center, Los Angeles, CA USA; ^8^ Department of Pediatrics, Division of Infectious Diseases and Immunology, Cedars‐Sinai Medical Center, Los Angeles, CA USA; ^9^ Department of Neurology, Cedars‐Sinai Medical Center, Los Angeles, CA USA; ^10^ Department of Biomedical Sciences, Division of Applied Cell Biology and Physiology, Cedars‐Sinai Medical Center, Los Angeles, CA USA

## Abstract

**Background:**

Alzheimer’s disease (AD) is a progressive irreversible dementia characterized by beta‐amyloid protein plaque deposition and hyperphosphorylation of tau forming neurofibrillary tangles, and neurodegeneration. An emerging theory posits that infections could be one of the triggering factors in AD development and progression. Multiple lines of evidence have linked *Chlamydia pneumoniae* (Cp), a gram‐negative obligate intracellular bacterium with AD. Cp has been detected in the post‐mortem brain tissues of AD patients, however, pathomechanisms associated with Cp in AD remain unknown.

**Method:**

Transgenic APP_SWE_/PS1_∆E9_ (ADtg) mice and non‐tg wildtype (WT) littermates were infected with Cp intranasally and sacrificed either 1 week or 6 months after infection. Mice were perfused with saline, and the brains were harvested for subsequent analysis, including PCR, flow cytometry, immunofluorescence, and live Cp growth. Neurobehavioral functions were assessed through an open field, visual‐stimuli X maze, and Barnes maze tests. Primary microglia and astrocytes were infected with Cp and cytokine and chemokines production was measured by ELISA.

**Result:**

We found Cp in the olfactory bulb and brain of mice 7 days after infection and the infiltration of inflammatory cells into the brain. Cp infection resulted in microglial activation. Immunohistochemical analysis also revealed the activation of microglia and astrocytes in the Cp‐infected mouse brain. In addition, Cp infection led to increased mRNA expression of neuroinflammatory mediators such as cytokines and chemokines. Primary astrocytes and microglia cultures could sustain Cp growth and produce proinflammatory cytokines and chemokines in response to infection. Live Cp in the brain was also observed 7 months after infection and the Cp load was higher in ADtg mice than WT. Cp infection resulted in significant cognitive decline in both WT as well as ADtg mice together with increased AD‐related neuropathology.

**Conclusion:**

Intranasal Cp infection leads to brain colonization and altered Iba1 immunoreactivity. Both primary astrocytes and microglia support Cp growth and produce neuroinflammatory cytokines. Long‐term Cp infection promotes neuroinflammation and cognitive decline in mice along with increased Aβ deposits. Cognitive impairment in Cp‐infected ADtg and WT mice as well as increased Aβ in ADtg mice suggest that Cp infection may play an important pathological role in AD development.

